# Extracellular Vesicles Promote the Formation of Pre-Metastasis Niche in Gastric Cancer

**DOI:** 10.3389/fimmu.2022.813015

**Published:** 2022-01-31

**Authors:** Diya Tang, Shanshan Liu, Hong Shen, Gongping Deng, Shan Zeng

**Affiliations:** ^1^ Department of Oncology, Xiangya Hospital, Central South University, Changsha, China; ^2^ National Clinical Research Center for Geriatric Disorders, Xiangya Hospital, Central South University, Changsha, China; ^3^ Department of Emergency, Hainan General Hospital, Hainan Affiliated Hospital of Hainan Medical University, Haikou, China

**Keywords:** gastric cancer, extracellular vesicles, immunomodulation, pre-metastatic, microenvironment

## Abstract

Globally, gastric cancer (GC) ranks fourth in the incidence of malignant tumors. The early clinical manifestations of GC lack specificity. Most patients are already at an advanced stage when they are first diagnosed, and their late progression is mainly due to peritoneal metastasis. A pre-metastatic microenvironment is formed, before the macroscopic tumor metastasis. Extracellular vesicles (EVs) are nanovesicles released by cells into body fluids. Recent studies have shown that EVs can affect the tumor microenvironment by carrying cargos to participate in cell-to-cell communication. EVs derived from GC cells mediate the regulation of the pre-metastasis niche and act as a coordinator between tumor cells and normal stroma, immune cells, inflammatory cells, and tumor fibroblasts to promote tumor growth and metastasis. This review highlights the regulatory role of EVs in the pre-metastatic niche of GC and mulls EVs as a potential biomarker for liquid biopsy.

## Introduction

Gastric cancer (GC) is one of the major global health problems. There are about one million new cases of GC reported all over the world yearly (1, 2). Most GC patients present with locally advanced or distant metastasis with an average overall survival of 10-12 months (3). Stage IIIC resected tumors carry a mere 5-year survival rate of 18%. Instead, the 5-year survival rates for stage IA and IB tumors treated with surgery are 94% and 88%, respectively (4). The pre-metastasis niche is a favorable microenvironment created by the primary tumor for the subsequent metastatic organs, following the famous “soil” and “seed” metastasis theory (5–7). According to the difference of biogenesis, EVs can be divided into microvesicles and exosomes. Their contents include nucleic acids, lipids, and proteins which can promote the malignant progression of cancer cells (8). GC-derived EVs play a critical role in tumor formation, proliferation, invasion, metastasis, drug resistance, and other processes. This article addresses the role of GC-derived EVs in the formation of pre-metastasis niches in GC.

## Biogenesis of EVs

**Figure 1:**
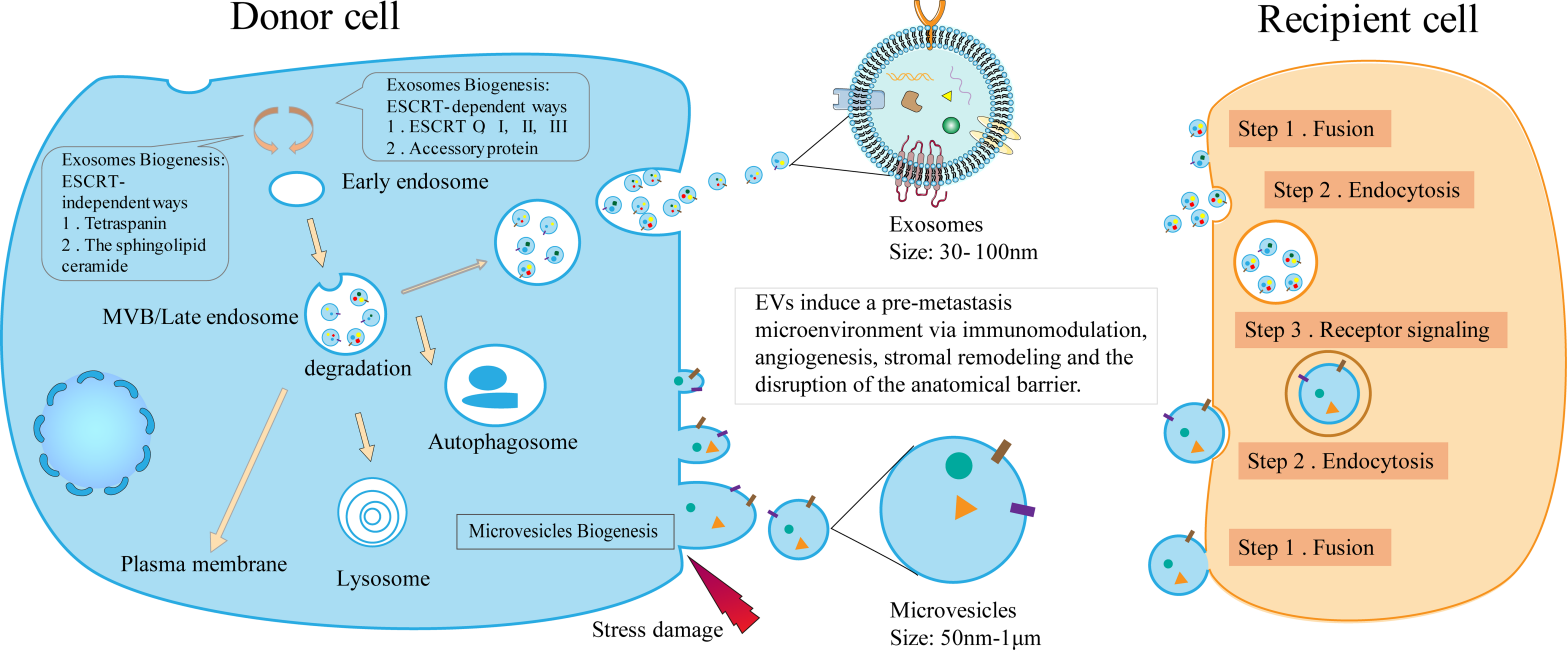
The biogenesis of EVs and the mechanism of the formation of pre-metastasis microenvironment. The biogenesis of EVs includes the occurrence of exosomes and microvesicles. By acting as communicators between donor and recipient cells, EVs induce immunomodulation, angiogenesis, stromal remodeling, and barrier disruption, ultimately contributing to the pre-metastasis microenvironment.

In a stable external environment, EVs can be released by all organisms (9). EVs are heterogeneous groups that, according to their biological origin, can be divided into two types: microvesicles and exosomes (10). The biogenesis, cargo assembly, secretion of exosome involves a set sequence of cellular events (**Figure 1**). Exosomes are nano-sized vesicles formed by early endosomes sprouting inward, leading to the generation and enrichment of intraluminal vesicles. It selectively contains proteins, nucleic acids, and lipids used to form advanced endosomes – multi vesicles body (MVBs). MVBs can be integrated with the plasma membrane, freeing the jammed intraluminal vesicle (ILV) (So-called exosomes) (10, 11). The endosomal sorting complex required for transport (ESCRT) is the chief driver of membrane shaping and splitting, and the conventional mechanism of MVBs and ILV formation (12, 13). ESCRT comprises four dissimilar protein complexes; ESCRT-0, -I, -II, -III, and auxiliary protein (14, 15). The utmost comprehensive research of ESCRTs in exosome biogenesis demonstrated that four in twenty-three ESCRT proteins affected exosomes’ secretion, and they include; HRS, TSG101, STAM1, and VPS4B protein (16). Interestingly, despite concurrent silencing of vital subunits of all four ESCRT-complexes, ILVs are still molded in MVBs (17). Therefore, it is possible to assume that exosomes can also be developed in an ESCRT-independent manner, like the tetraspanin family (CD63, CD81, CD83) and lipid metabolism enzymes neutral sphingomyelinase (nSMase) (10). Compared to the biological generation of exosomes, microvesicles are somewhat more straightforward. New microvesicles are formed by the outward budding and contraction of the plasma membrane (11).

## GC-Derived EVs

Kagota et al. combined pretreatment technology with ultracentrifugation to separate and purify EVs from gastric juice of GC patients (18). The substances released by EVs included DNA, RNA, proteins, lipids, and metabolites (19). Extensive previous research has proved that the protein cargo of EVs can speed up the process of GC metastasis (**Table 1**) (20–24). For example, by detecting the expression of TGF-β1 in exosomes isolated from the gastric omental veins of 61 GC patients, as well as regulatory T cells (Treg) in lymph nodes of GC. The researchers found that the expression of TGF-β1 was closely related to the pathological stage, lymph node metastasis, and the proportion of Treg cells. In addition, exosomes derived from GC patients act on TGF-β1, which can induce Treg cell formation (25). Ectopic expression of exosomes has been observed, whereby the EGFR expressed by exosomes isolated from GC cells not only enters the liver but also integrates into the plasma membrane of liver stromal cells. Also, translocation of EGFR promoted tumor invasion and metastasis by hindering miR-26a/b, thus, up-regulating the expression of hepatocyte growth factor (HGF) (26).

**Table 1:** Summary of proteins in EVs involved in GC progression and metastasis.

Extracellular vesicles proteins	Recipient cells/Target	Role/Mechanism
CD97	NA	Lymphatic metastasis (20)
CD97	MAPK signaling pathway	Proliferation and Invasion (21)
FZD10	NA	Carcinogenesis and Tumor proliferation (22)
FZD10	Wnt signaling pathway	Cancer progression (23)
MET	TAMs	Cancer progression (24)
TGF-β1	Treg cells	Lymphatic metastasis (25)
EGFR	HGF	Liver metastasis (26)

Evs, Extracellular vesicles; EGFR, Epidermal growth factor receptor; FZD10, Frizzled10; GC, Gastric cancer; HGF, Hepatocyte growth factor; TAMs, Tumor-associated macrophages; Treg, Regulatory T; TGF-β1, Transforming growth factor β1; Wnt, Wingless/Integrated; MAPK, Mitogen-activated protein kinase; MET, Mesenchymal-epithelial transition factor NA, Not available.

Apart from proteins, nucleic acids also play a vital role in the metastasis of GC. For instance, when exosomes were isolated from GC cells and gastric mucosal epithelial cells, the expression level of miR-155-5p in GC- derived exosomes were significantly increased. Simultaneously, the expression of TP53INP1 protein in GC cells was down-regulated, which confirmed that TP53INP1 was directly affected by miR-155-5p regulation. More importantly, when GC cell line AGS was cultured with isolated exosomes rich in miR-155-5p, the proliferation and migration ability of AGS cells was increased, confirming that exosome miR-155-5p facilitated the invasion and metastasis in GC by directly acting on TP53INP1 (27). *In vitro* experiments showed that GC-derived exosomes can be absorbed by peritoneal mesothelial cells and further up-regulate the expression of miR-21-5p and directly target SMAD7 to promote peritoneal metastasis. The researchers also established a mouse tumor peritoneal diffusion model, verifying that exosome miR-21-5p induces peritoneal mesothelial cells (PMCs) mesothelial-to-mesenchymal transition (MMT) and promote cancer to the peritoneum by targeting SMAD7 (28). After co-cultivation of umbilical vein endothelial cells and GC-derived exosomes, the expression of miR-23a was up-regulated and directly targeted PTEN to promote tumor angiogenesis. Furthermore, VEGF was up-regulated, and TSP-1 was down-regulated (29). Collectively, the nucleic acid cargo of EVs regulates the gene expression of recipient cells not only locally but also in a systemic manner. Also, in the initial stage of malignant tumors, it promotes the formation of the pre-metastasis microenvironment and subsequent migration (**Table 2**) (30–48).

**Table 2:** Summary of non-coding RNAs in EVs involved in GC progression and metastasis.

Non-coding RNAs	Recipient cells/Target	Role/Mechanism
miR-155-5p	TP53INP1 mRNA	Proliferation and Migration (27)
miR-21-5p	PMCs	Peritoneal metastasis (28)
miR-23a	HUVECs/PTEN	Angiogenesis (29)
lncRNA PCGEM1	NA	Invasion and Metastasis (30)
miR-106a	Smad7	Peritoneal metastasis (31)
miR-15b-3p	DYNLT1/Caspase-3/Caspase-9 Signaling Pathway	Tumorigenesis and Malignant transformation (32)
miR-155	Endothelial Cells/c-MYB/VEGF Axis	Angiogenesis (33)
miR-155	Endothelial Cells/Forkhead Box O3	Angiogenesis (34)
miR-196a-1	SFRP1	Invasion and Metastasis (35)
miR-1290	NKD1	Proliferation and Invasion (36)
miR-135b	Endothelial Cells/FOXO1 Expression	Angiogenesis (37)
miR-501	BLID	Chemoresistance and Tumorigenesis (38)
miRNA expression profiles analysis: 29 miRNA were identified	NA	Peritoneal dissemination (39)
miR-423-5p	SUFU	Cancer growth and Metastasis (40)
miR-130a	Vascular endothelial Cells/Targeting C-MYB	Angiogenesis (41)
miR-27a	Fibroblasts/CSRP2	Proliferation, Motility, and Metastasis (42)
lncRNA** **ZFAS1	NA	Progression (43)
miR-217	CDH1	Carcinogenesis (44)
Let-7 microRNA	RAS and HMGA2	Tumorigenesis and Metastasis (45)
miR-21 and miR-1225-5p	NA	Invasion (46)
circNRIP1	AKT1/mTOR pathway	Progression (47)
circ-RanGAP1	miR-877-3p	Invasion and Metastasis (48)

BLID, BH3-like motif-containing protein; CDH1, Cadherin-1; Evs, Extracellular vesicles; GC, Gastric cancer; HUVECs, Human umbilical vein endothelial cells; HMGA2, high mobility group AT-hook 2; mTOR, Mammalian target of rapamycin; NA, Not available; PMCs, Peritoneal mesothelial cells; PTEN, Phosphatase and tensin homolog; SUFU, Suppressor of fused protein; SFRP1, Secreted frizzled-related protein 1; VEGF, vascular endothelial growth factor.

## Roles of GC-Derived EVs in Remodeling the Pre-Metastasis Niche of GC

**Figure 2:**
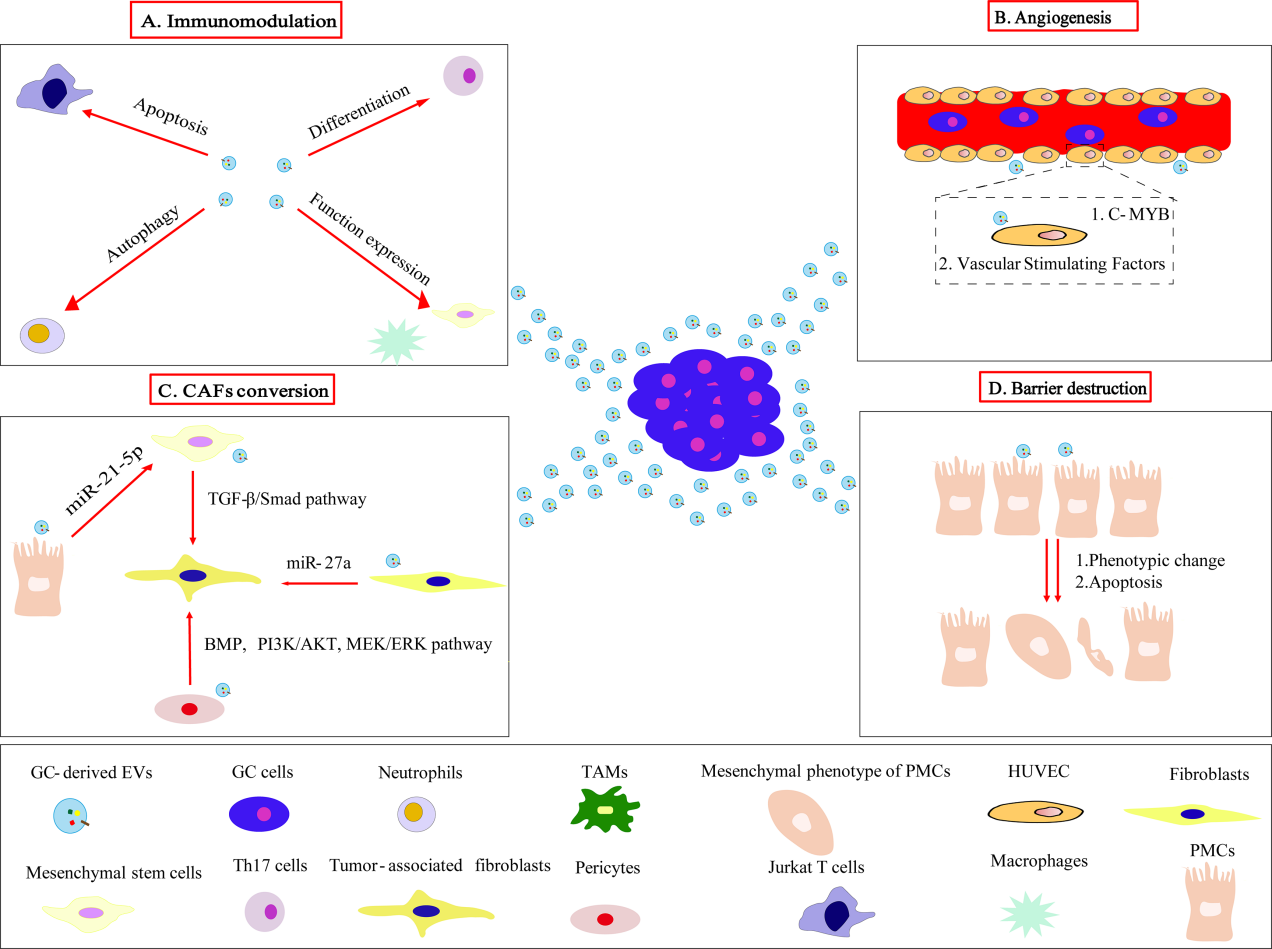
GC-derived EVs acts on target cells to mediate the formation of pre-metastasis niche. **(A)** GC-derived EVs modulate the immune microenvironment by inducing Jurkat T cells apoptosis, neutrophils autophagy, Th17 cells differentiation, functional expression of macrophages and mesenchymal stem cells, eventually promoting tumor progression and metastasis. **(B)** GC-derived EVs target c-MYB on vascular endothelial cells and regulate vascular stimulating factors to promote angiogenesis. **(C)** GC-derived EVs convert fibroblasts, pericytes, mesenchymal stem cells, PMCs to CAFs through different mechanisms. **(D)** Phenotypic changes and apoptosis of PMCs induced by GC-derived EVs clear the peritoneal mesothelial cell barrier layers.

We summarized the roles of GC-derived EVs in reshaping the pre-metastasis niche of GC which promotes the colonization and survival of circulating tumor cells after reaching the secondary site (**Figure 2**).

## Immunomodulation

The pre-metastasis niche creates a favorable microenvironment for circulating tumor cells to metastasize to specific organs and locations (49). The process of tumor metastasis is closely related to the body’s immune system (50). EVs derived from GC act on specific signaling pathways to induce immune cell apoptosis, autophagy, and functional expression to promote tumor progression (51). In the setting of adaptive immunity, Qu et al. demonstrated that GC-derived exosomes could increase PI3K proteasome degradation through the ubiquitin ligase cbl family, inactivate PI3K/Akt signaling, and activate caspase3, 8, 9 to mediate apoptosis of Jurkat T cells (52). However, in the innate immune response, GC-derived exosomes induced neutrophils autophagy and promoted tumor activation by acting on HMGB1/TLR4/NF-κB signals (53). GC-derived exosomes can induce macrophages to secrete inflammatory factors through the NF-κB signaling pathway to promote tumor proliferation, migration, and invasion (54). Besides, under the hypoglycemic state, the expression of miR-451 carried by GC-derived exosomes was up-regulated, which promoted Th17 cells differentiation by activating the NF-κB pathway (55). Previous studies have shown that mesenchymal stem cells are also related to immune regulation. When Shen et al. co-cultured mesenchymal stem cells with GC-derived exosomes, they found that mesenchymal stem cells can activate immune cells through the NF-κB signaling pathway, maintain the function of the inflammatory environment, thereby promoting tumor growth (56). All the aforementioned studies indicate that GC-derived exosomes play a negative regulatory role by acting on immune cells, thereby helping to form the pre-metastatic niche before metastasis (**Supplementary Table 1**).

## Angiogenesis

Angiogenesis refers to the process of forming new blood vessels from the original capillaries or post-capillary veins (57). These new capillaries provide the tumor with nutrients, oxygen, and growth factors to promote tumor growth and metastasis (58). GC-derived EVs form a pre-metastatic microenvironment that is conducive to tumor growth by promoting tumor angiogenesis. Previous studies have shown that C-MYB, as a transcription factor, is closely related to angiogenesis. Researchers have confirmed that human umbilical vein endothelial cells (HUVECs) that overexpressed c-MYB have reduced migration, proliferation, and ring formation. GC-derived exosomes also express miR-130a, which promotes angiogenesis by targeting C-MYB on vascular endothelial cells, whether *in vivo* or *in vitro* (41). After the researchers incubated vascular endothelial cells with exosomes derived from irradiated GC cells, the proliferation, mobility, and invasiveness of vascular endothelial cells increased which was counteracted by VEGFR-2 inhibitor- Apatinib (59). These studies suggest that GC-derived EVs promote vascular endothelial cells proliferation and migration in different ways to achieve angiogenesis.

## Stromal Remodeling

Stromal cells and extracellular matrix components are the key components of the tumor microenvironment, providing support and nutrition in tumor growth, progression, and metastasis (60). The stromal cells mainly include fibroblasts, inflammatory cells, immune cells, and pericytes. EVs secreted by tumors play a crucial role in stromal cell remodeling by reprogramming fibroblasts, pericytes, immune cells, and by extension, mediating the formation of an extracellular matrix that facilitates the growth of secondary tumor sites, thereby promoting tumor growth, invasion, and metastasis (12).

### Cancer-Associated Fibroblasts (CAFs) Conversion

Among all the stromal cells, CAFs play a crucial role in tumor invasion, progression, and metastasis (61). By secreting a series of chemokines, cytokines, and proteases to participate in matrix remodeling, regulating the recruitment and function of immune cells in the tumor microenvironment; hence creating a niche for cancer cells to promote their malignant tendencies (62). EVs mediate the transformation of stromal cells into CAFs by acting as a conduit between tumor cells and stromal cells (60). Proofs have shown that high and stable expression of miR-27a in exosomes was related to poor clinical prognosis. Similarly, GC-derived exosomes mediated the transformation of fibroblasts into CAFs by expressing miR-27a, and have been shown to promote the proliferation and metastasis of cancer cells both *in vivo* and *in vitro* (42). Similarly, Wang et al. postulated that GC-derived exosomes can induce macrophages to differentiate into PD1 + tumor-associated macrophages, thereby creating an environment for tumor progression (63). Moreover, GC-derived exosomes can promote the proliferation and migration of pericytes by inducing the transformation of pericytes into CAFs by transferring bone morphogenetic proteins (BMP) and activating PI3K/AKT and MEK/ERK pathways (64). Mesenchymal stem cells have great potential for self-renewal and multi-directional differentiation. Studies have shown that exosomes derived from GC mediate the transformation of human umbilical cord-derived mesenchymal stem cells into CAFs by transferring TGF-β and activating TGF-β/Smad pathway (65). It is particularly worth mentioning that PMCs are also one of the important sources of CAFs. On the one hand, when EVs are not involved, the study on peritoneal metastasis has shown that normal mesothelial cells can be converted into CAFs through MMT (66). On the other hand, for EVs, PMCs can be transformed into mesenchymal stem cells first and then into CAFs. GC-derived EVs mediate the transformation of PMCs into mesenchymal cells (67), for example, PMCs can internalize GC-derived exosomes miR-21-5p, and induce PMC MMT by activating the TGF-β/Smad pathway *via* targeting SMAD7 (28). And the mechanism of transition of mesenchymal stem cells to CAFs is as described previously. Besides, another study has shown that EVs derived from malignant ascites may promote the transformation of PMCs to CAFs through TGF-β1-induced MMT, and in the EVs-treated group, the phenotypic changes of PMCs under morphological and western blotting assays all indicated a CAFs-like transition (68). Lastly, before metastasis to secondary organs, GC-derived EVs mediate the transition of normal stroma to cancer stroma.

### CAFs and Tumor-Associated Macrophages (TAMs) Function

GC-derived exosomes promote tumor growth, invasion, and metastasis by directly suppressing immune responses and inducing the formation of CAFs and TAMs phenotypes (69). GC-derived EVs promote the transformation of various cells, including fibroblasts, pericytes, PMCs and mesenchymal stem cells, into CAFs with a malignant phenotype (70). Evidence has shown that CAFs promote the migration and invasion of GC cells, and enhance angiogenesis and metastasis that are essential for tumor cells colonization, survival, and growth (71–75). Previous studies have shown that CAFs increased GC cell mobility and invasiveness by up-regulating Rhomboid 5 Homolog 2 (RHBDF2) expression through the TGF-β1 signaling pathway (76). Ding et al. showed that CAFs mediate GC angiogenesis through the PI3K/AKT and ERK1/2 signaling pathways through the expression of HGF (77). In addition, CAFs also mediate the invasion and metastasis of GC by acting on multiple signal pathways. For example, Bae’s study showed that inhibiting the GAS6/AXL axis can block the interaction between CAFs and GC cells, thereby inhibiting tumor progression (78). CAFs can promote GC in a ligand-independent manner by activating erythropoietin-producing hepatocyte A2 (EphA2) (79). CAFs also activate CXCL12/CXCR4 to promote integrin b1 aggregation and invasiveness in GC (80). Kurashige’s research showed that cancer-related epigenetic regulation and inhibition of miR-200b by fibroblasts contributes to cancer infiltration and peritoneal spread of GC (81). Activated GC-associated fibroblasts promote the malignant phenotype and 5-FU of GC through paracrine drug resistance (82). CAFs can promote the malignant degree of GC cells through nodal signaling (83). In addition, previous research has shown that CAFs-derived lumican promotes the progress of GC through the integrin b1-FAK signaling pathway (84).

In the pre-metastatic microenvironment, GC-derived EVs mediate the differentiation of local macrophages into immunosuppressive M2 macrophages. TAMs promote tumorigenesis *via* their exosome secretion. Study has shown that TAMs-derived exosomes promote migration of GC cells through the transfer of functional apolipoprotein E (85). In addition, TAMs play an important role in the invasion, development, angiogenesis and metastasis of GC. Evidence has proved that polarized CD163+ TAMs were associated with increased angiogenesis in GC (86). TAMs regulated the invasion and metastasis of GC cells through the TGFβ2/NF-κB/Kindlin-2 axis (87). TAMs may cause an increase in Bmi1 expression through miR-30e inhibition, leading to gastrointestinal tumor progression (88). TAMs induced forkhead box Q1 (FOXQ1) expression and promoted GC cell epithelial-mesenchymal transition and metastasis (89). Ding et al. confirmed that CCL5 secreted by TAMs may promote the proliferation, invasion, and metastasis of GC cells through the Stat3 signaling pathway (90). TAMs induce GC invasion and poor prognosis *via* cyclooxygenase2/MMP9 dependent manner (91). Lastly, macrophages in the peritoneum of GC patients play a supportive role in peritoneal metastasis by producing EGF and VEGF (92). All this evidence highlights that EVs mediate the transformation of normal stroma to cancer stroma, and blocking this transformation is essential for the distant metastasis of tumors.

## GC-Derived EVs Mediated Organophilic Metastasis

### Barrier Destruction and Peritoneal Metastasis (PM)

Most advanced GC metastasizes to the peritoneum. Therefore, it is crucial to understand the mechanism of PM. Stephen Paget’s “seed and soil” hypothesis offers great insight into understanding the mechanism of PM (93). The first line of defense for all abdominal metastasis is the mesothelial cell (94). Recent studies have shown that GC-derived EVs can break through the first barrier of PM by promoting mesothelial cell damage, apoptosis, and phenotypic changes. Deng et al. confirmed that GC-derived exosomes not only induced mesothelial cell apoptosis, but also mediated the transformation of mesothelial cells to mesenchyme, which leads to the destruction of the mesothelial barrier and peritoneal fibrosis, and ultimately promotes PM (67). Likewise, exosomal miR-21-5p induced the mesothelial to mesenchymal transition of peritoneal mesothelial cells by targeting Smad7 and promoted the peritoneal spread of cancer (28).

Research has also revealed that exosomes from malignant ascites in GC patients supported epithelial-mesenchymal transition (EMT) signaling in GC cells in the mouse peritoneal tumor model, thus, resulting in peritoneal tumor cell dissemination (39). Furthermore, the co-culture of exosomes derived from malignant pleural fluid of GC and GC cells can promote tumor migration and increase the expression of mesothelial cell adhesion-related molecules, such as fibronectin 1 and laminin gamma 1, which may be the mechanism of peritoneal metastasis (95). GC-derived EVs induced PMCs infiltration by activating Wnt3a/β -catenin signaling, infiltrating PMCs, and in turn, promoting the subserosal invasion of cancer cells, and mutual attraction between cancer cells and PMCs accelerated tumor invasion which ultimately led to peritoneal metastasis (96). Besides, exosomes derived from GC can mediate peritoneal dissemination *via* participating in the regulation of signaling pathways. There is evidence that the delivery of miR-106a from GC-derived exosomes plays a vital role in GC peritoneal metastasis *via* direct regulation of Smad7 (31). Incubation of GC cells with peritoneal mesothelial HMrSV5 cells showed that miR-544 could be transferred from GC-derived EVs to peritoneal cells, where it suppresses the promyelocytic leukemia zinc finger expression and ultimately leads to peritoneal metastasis of GC (97). Zhu et al. proposed that Nicotinamide N-methyltransferase-containing exosomes derived from GC cells could promote peritoneal metastasis *via* TGF-β/smad2 signaling (98).

### Liver Metastasis (LM)

In addition to PM, GC-derived EVs are also involved in the LM of GC. Highly aggressive GC cells can secrete exosomes containing miR-196a-1, which may be related to liver metastasis *in vitro* and *in vivo* by targeting SFRP1 (35). In addition, the study also showed that EGFR in exosomes secreted from GC cells can be delivered into the liver and is integrated into the plasma membrane of liver stromal cells, the translocated EGFR effectively activates HGF by inhibiting the expression of miR-26a/b. Secondly, the upregulated paracrine HGF binds to the c-MET receptor on the migrated cancer cells, which was conducive to the formation of a hepato-like microenvironment that promotes liver-specific metastasis (26). In summary, there is increasing evidence on the role of GC-derived EVs in organophilic metastasis.

## EVs as Biomarkers

Stable, simple, relatively non-invasive, easy to monitor and follow-up, and tumor specificity are the special advantages of EVs as biomarkers. Previous studies have revealed that exosomal miR-23b can be used as a potential minimally invasive predictive biomarker, suitable for detecting recurrence and prognosis in all GC patients irrespective of stages (99). Previous studies have also demonstrated that exosomal HOXA transcript at the distal tip (HOTTIP) could be a potential biomarker in GC diagnosis and prognosis (100). Recent research has shown that low expression of miR-29b in peritoneal exosomes was associated with postoperative peritoneal recurrence (101). Similarly, a study showed that mesenchymal stem cells derived from GC tissues promoted the progression of GC by transferring exosomal miRNAs to GC cells, thereby providing a potential biomarker for GC (102). Finally, exosomes also promote the progression of GC by expressing proteins. For instance, a previous study showed that the overall survival rate of the high exosomal PD-L1 group was significantly lower than the low exosomal PD-L1 group, exosomal PD-L1 in GC patients was negatively associated with CD4 T cell and CD8 T-cell count, indicating that exosomal PD-L1 was associated with the immunosuppressive status of GC patients (103). In addition, previous research has shown that CD63 is one of the possible prognostic indicators for patients with GC, since CD63-positive exosomes may be related to the interaction between stromal cells and cancer cells (104).

## Conclusion

GC-derived EVs promote the formation of the microenvironment before metastasis through immunomodulation, angiogenesis, stromal cell remodeling, barrier destruction, and organophilic metastasis. Initially, the pre-metastasis microenvironment heralded a lot of excitement in the field of cancer research. With the recent advancement of research, we continue to understand the vital role of EVs in the pre-metastasis microenvironment. At present, the isolation and purification of EVs *in vitro* are not complicated. Therefore, it should be harnessed as an available and cost-effective potential biomarker in early cancer detection, treatment evaluation, and follow-up. New technologies, including liquid biopsy, are moving the field forward in understanding the mechanisms of tumor metastasis. The latest developments make EVs a promising new field for early diagnosis and detection across cancer types.

## Author Contributions

DT was a major writer of the manuscript and designed the tables. GD and SZ conceived and designed the article and guided the selection of references. SL and HS were responsible for figure design and language modification. All the authors read and approved the final manuscript.

## Funding

This study was supported by a grant of the National Key R & D Program of China (No. 2018YFC1313300); six grants from the National Natural Science Foundation of China (No. 81070362, 81172470, 81372629, 81772627, 81874073 & 81974384); two projects from the Nature Science Foundation of Hunan Province (No. 2021JJ31092 & 2021JJ31048), and a project from China Cancer Elite Team Innovative Grant (No. 201606).

## Conflict of Interest

The authors declare that the research was conducted in the absence of any commercial or financial relationships that could be construed as a potential conflict of interest.

## Publisher’s Note

All claims expressed in this article are solely those of the authors and do not necessarily represent those of their affiliated organizations, or those of the publisher, the editors and the reviewers. Any product that may be evaluated in this article, or claim that may be made by its manufacturer, is not guaranteed or endorsed by the publisher.

## Abbreviations

ApoE, Apolipoprotein E; BMP, Bone morphogenetic proteins; BLID, BH3-like motif-containing protein; CAF, Cancer-associated fibroblasts; CD97, Recombinant Cluster Of Differentiation 97; CDH1, Cadherin-1; EGFR, Epidermal growth factor receptor; EphA2, Erythropoietin-producing hepatocellular A2; EMT, Epithelial-mesenchymal transition; ESCRT, The endosomal sorting complex required for transport; EVs, Extracellular vesicles; FOXQ1, Forkhead box Q1; FZD10, Frizzled10; GC, Gastric cancer; HGF, Hepatocyte growth factor; HOTTIP, The HOXA transcript at the distal tip may; HUVECs, Human umbilical vein endothelial cells; HMGA2, High mobility group AT-hook 2; IVL, Intraluminal vesicle; MIA, Melanoma inhibitory activity; MMT, Mesothelial-to-mesenchymal transition; MVBs, Multivesicular body; MAPK, Mitogen-activated protein kinase; mTOR, Mammalian target of rapamycin; nSMase, Lipid metabolism enzymes neutral sphingomyelinase; PMCs, Peritoneal mesothelial cells; PM, Peritoneal metastasis; PTEN, Phosphatase and tensin homolog; RHBDF2, Rhomboid 5 Homolog 2; SUFU, Suppressor of the fused protein; SFRP1, Secreted frizzled-related protein 1; TAMs, Tumor-associated macrophages; TGF-β, Transforming growth factor β; TGF-β1, Transforming growth factor β1; Treg, Regulatory T; VEGF, Vascular endothelial growth factor; Wnt, Wingless/Integrated.
