# Supplementary material for: Extracellular Vesicles Promote the Formation of Pre-Metastasis Niche in Gastric Cancer
Source: Front Immunol. 2022 Jan 31;13:813015. doi: 10.3389/fimmu.2022.813015 (PMC8841609; doi:10.3389/fimmu.2022.813015)
Supplement: Supplementary file 1 [file Table_1.docx]

**Suppl. Table 1. Roles of GC-derived EVs in promoting the pre-metastasis niche of GC.**

| ***Evs Function*** | ***Recipient cells*** | ***Effect*** | ***Potential targets/pathways*** |
| --- | --- | --- | --- |
| Immunomodulation | Jurkat T cells | Apoptosis | The ubiquitin ligase cbl family, PI3K / Akt signaling,caspase3, 8, 9^[52]^ |
|  | Neutrophils | Autophagy | HMGB1 / TLR4 / NF-κB signaling^[53]^ |
|  | Th17 cells/ macrophages/ mesenchymal stem cells | Differentiation/ function expression | NF-KB pathway^[54-56]^ |
| Angiogenesis | HUVEC | Upregulate miR-130a | c-MYB^[41]^ |
|  | HUVEC | proliferation, motility, and invasiveness | Vascular stimulating factors^[59]^ |
| Stroma remodeling | Fibroblasts | CAFs | miR-27a^[42]^ |
|  | Macrophages | PD1+ TAMs | Inhibit the function of CD8 T cells ^[63]^ |
|  | Pericytes | CAFs | BMP, PI3K / AKT, MEK / ERK pathway^[64]^ |
|  | Mesenchymal stem cells  PMCs | CAFs  CAFs | TGF-β/Smad pathway^[65]^  miR-21-5p,Smad7 ^(28)^ |
| Barrier destruction | mesothelial cell | Apoptosis and phenotypic changes | NA^[67]^ |
|  | mesothelial cell | Phenotypic changes | miR-21-5p,Smad7^[28]^ |
|  | mesothelial cell | Phenotypic changes | EMT^[39]^ |
|  | mesothelial cell | Increased adhesion molecules | FN1, LAMC1^[95]^ |
|  | mesothelial cell | Infiltration | Wnt3a/β-catenin signaling^[96]^ |
| Organophilic metastasis | mesothelial cell | PM | Smad7^[31]^ |
|  | mesothelial cell | PM | PLZF^[97]^ |
|  | mesothelial cell | PM | TGF-β/smad2 signaling^[98]^ |
|  | low-invasive GC cells | LM | SFRP1^[35]^ |
|  | liver stromal cells | LM | HGF/ miR-26a/b, c-MET^[26]^ |

***Abbreviations:*** *CAF* Cancer-associated fibroblasts, *Evs* Extracellular vesicles, *EMT* Epithelial-mesenchymal transition, *FN1* Fibronectin 1, *GC* Gastric cancer, *HGF* Hepatocyte growth factor, *HUVEC* Human umbilical vein endothelial cell, *LM* Liver metastasis, *LAMC1* Laminin gamma 1, *NA* Not Available, *PM* Peritoneal metastasis, *PMCs* Peritoneal mesothelial cells, *PLZF* Promyelocytic leukemia zinc finger, *SFRP1* Secreted frizzled-related proteins, *TAMs* Tumor-associated macrophages, *TGF-β* Transforming growth factor β.
